# Wwox Binding to the Murine Brca1-BRCT Domain Regulates Timing of Brip1 and CtIP Phospho-Protein Interactions with This Domain at DNA Double-Strand Breaks, and Repair Pathway Choice

**DOI:** 10.3390/ijms23073729

**Published:** 2022-03-28

**Authors:** Dongju Park, Mehdi Gharghabi, Colleen R. Reczek, Rebecca Plow, Charles Yungvirt, C. Marcelo Aldaz, Kay Huebner

**Affiliations:** 1 Department of Cancer Biology and Genetics, The Comprehensive Cancer Center, The Ohio State University Wexner Medical Center, Columbus, OH 43210, USA; mehdi.gharghabi@gmail.com (M.G.); plow.3@buckeyemail.osu.edu (R.P.); yungvirt.2@buckeyemail.osu.edu (C.Y.); 2 Department of Outcomes and Translational Sciences, College of Pharmacy, The Ohio State University, Columbus, OH 43210, USA; 3 Department of Pathology & Cell Biology, Columbia University Irving Medical Center, Columbia University, New York, NY 10032, USA; colleenreczek@northwestern.edu; 4 Department of Epigenetics and Molecular Carcinogenesis, The University of Texas MD Anderson Cancer Center, 1881 East Road, Houston, TX 77054, USA; maaldaz@mdanderson.org

**Keywords:** end-resection at DSBs, Wwox deficiency, Chk2 inhibition, IR and platinum treatment resistance

## Abstract

Wwox-deficient human cells show elevated homologous recombination, leading to resistance to killing by double-strand break-inducing agents. Human Wwox binds to the Brca1 981-PPLF-984 Wwox-binding motif, likely blocking the pChk2 phosphorylation site at Brca1-S988. This phosphorylation site is conserved across mammalian species; the PPLF motif is conserved in primates but not in rodents. We now show that murine Wwox does not bind Brca1 near the conserved mouse Brca1 phospho-S971 site, leaving it open for Chk2 phosphorylation and Brca1 activation. Instead, murine Wwox binds to Brca1 through its BRCT domain, where pAbraxas, pBrip1, and pCtIP, of the A, B, and C binding complexes, interact to regulate double-strand break repair pathway response. In Wwox-deficient mouse cells, the Brca1-BRCT domain is thus accessible for immediate binding of these phospho-proteins. We confirm elevated homologous recombination in Wwox-silenced murine cells, as in human cells. Wwox-deficient murine cells showed increased ionizing radiation-induced Abraxas, Brca1, and CtIP foci and long resected single-strand DNA, early after ionizing radiation. Wwox deletion increased the basal level of Brca1-CtIP interaction and the expression level of the MRN-CtIP protein complex, key players in end-resection, and facilitated Brca1 release from foci. Inhibition of phospho-Chk2 phosphorylation of Brca1-S971 delays the end-resection; the delay of premature end-resection by combining Chk2 inhibition with ionizing radiation or carboplatin treatment restored ionizing radiation and platinum sensitivity in Wwox-deficient murine cells, as in human cells, supporting the use of murine in vitro and in vivo models in preclinical cancer treatment research.

## 1. Introduction

Expression of the Wwox tumor suppressor protein, with highly conserved amino acid sequences across species from fish to man [1], is frequently downregulated in human and mouse cancer genomes through deletion, translocation, and promoter hypermethylation, due to its location at a common chromosome fragile site [2]. Reduced Wwox expression is associated with cancer development and poor outcome in human cancers [3,4,5,6] and has been implicated in the DNA damage response [7,8,9,10]. We described a role for Wwox in the modulation of a double-strand break (DSB) repair pathway choice in human cancer cells, such that Wwox-deficient cells showed increased homologous recombination (HR) repair immediately following DNA damage, and resistance to killing by some DSB-inducing agents. Parallel experiments in murine cells revealed that Wwox knockout (KO) mouse embryonic fibroblasts (MEFs) also exhibited cytogenetic instability and resistance to ionizing radiation (IR) [7]. 

DSBs are deleterious DNA lesions, which are repaired through two major pathways: HR and non-homologous end joining (NHEJ). HR is considered a faithful repair pathway, preferentially occurring during late S and G2 phase in which the sister chromatid is used as the repair template. In contrast, NHEJ directly ligates broken DNA ends, causing small insertions or deletions. In addition to HR, DSBs can be repaired by alternative end-joining (Alt-EJ) and single-strand annealing (SSA); Alt-EJ joins the broken ends by aligning flanking microhomologous sequences and SSA anneals DSBs between repeat sequences, causing deletion and rearrangement [11,12]. 

HR is orchestrated by the involvement of Brca1 [13,14,15,16,17,18,19], which recruits large protein complexes to its BRCT domain (Brca1 C-terminal) to execute distinct functions with specific timing in response to induction of DSBs. The tandem repeat BRCT domain (tBRCT) directly interacts with at least three phosphorylated proteins, Abraxas, Brip1 (aka Bach1/FANCJ), and CtIP (hereafter referred to as Abraxas, Brip1, CtIP), to form the A, B, C complexes, which are implicated with Brca1, in cell cycle regulation, specific DNA repair functions and tumor suppression [20,21,22,23,24,25,26,27]. Mutations targeting the BRCT tandem repeats occur as causes of familial breast cancers and are thus clinically relevant, with experimental evidence showing that mutations disrupting BRCT pSPXF-binding interactions at the groove between the two repeats, either engineered (K1702M) or naturally occurring in familial breast cancers (M1775R), were associated with excessive end-resection and faulty hyperrecombination [28,29], underlining the importance of the tBRCT domain in maintaining genome integrity. 

The Mre11-Rad50-Nbs1 (MRN)-CtIP complex is involved in initiation of DNA end-resection at DSBs, which generates 3′ overhang single-strand DNA (ssDNA), and the timing of end-resection dictates the choice of DSB repair pathways [23,30,31,32]. CtIP is essential for the MRN-induced nicking step that initiates DNA resection. Though some previous studies have indicated that Brca1-CtIP interaction is not always required for end-resection [33,34], disruption of the Brca1-C complex reduces end-resection activity [35], implying that modulation of resection by Brca1-CtIP interaction affects DSB repair choice. 

During DNA damage repair, multiple serine residues in Brca1 are phosphorylated mainly by ATM/ATR and Chk2, which is critical for Brca1 functions [14,36,37]. Chk2 phosphorylates human Brca1 at Ser988, analogous to Ser971 in murine Brca1, initiates activation of Brca1, and affects timing of HR-mediated repair events such as DNA end-resection and Brca1 degradation [16,38]. In previous studies, we reported Brca1-Wwox interaction in human cells and elevated HR/SSA in Wwox-silenced human cells, leading to resistance to killing by DSB-inducing agents [7,10].

The current study was focused on determining how Wwox expression prevents this early initiation of HR in Wwox-positive MEF cell lines. Given that mouse strains are frequently used in designing and testing preclinical cancer treatment models, we sought to identify Wwox binding regions in murine Brca1 and associated proteins, to define molecular details of its role in DSB repair, in cooperation with Brca1.

## 2. Results

### 2.1. Wwox Binds to Brca1 through the BRCT Domain

**Figure 1:**
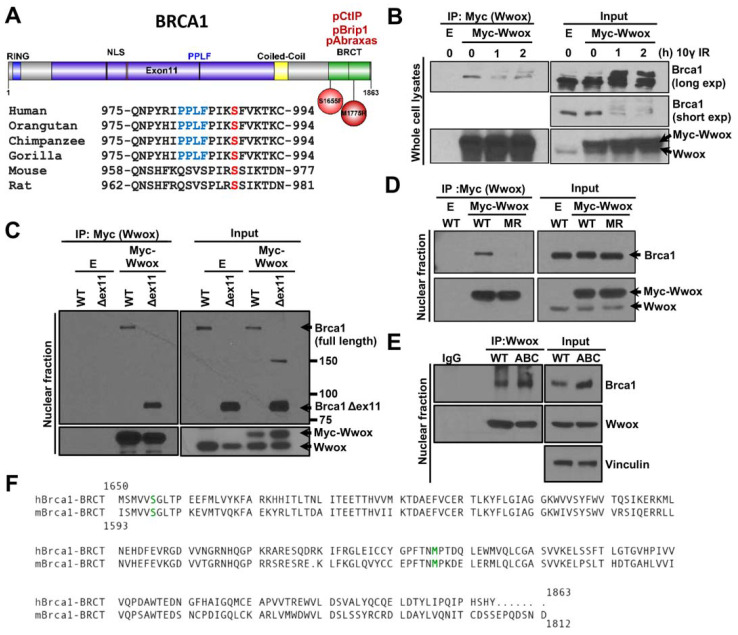
Mouse Wwox binds Brca1, likely through the BRCT domain. (**A**) Amino acid sequence alignment of Brca1 loci of primate and rodent species near mouse S971 and human S988 in exon 11, serines critical for activation of the HR repair pathway conserved across mammalian species; the PPLF motif, the primate Wwox-binding site, is not conserved in rodent Brca1. (**B**) Transfected Myc-Wwox co-immunoprecipitated with mouse Brca1. Transfected Myc-Wwox in MEFs pulled down both non-phospho and IR induced-phospho-Brca1. Note: Brca1 upshift after IR. (**C**) Brca1 exon 11, which is essential for Wwox-Brca1 interaction in human cells, is dispensable for Wwox-Brca1 interaction in MEFs. Both WT and Δexon11 (Δex11) Brca1 co-immunoprecipitated with Myc-Wwox in MEFs. (**D**) Brca1 BRCT domain mutant M1717R (MR) failed to interact with Wwox. E is empty vector transfected negative control. (**E**) Interaction of the pAbraxas, pBrip1, and pCtIP with BRCT domain in Brca1 is dispensable for Wwox-Brca1 binding, as shown by Wwox binding in ABC mutant cells, in which those three phospho-ligands do not bind the BRCT domain. (**F**) The human and mouse Brca1 BRCT domains are aligned, with sites of the SF and MR mutants that prevent binding of the pAbraxas, pBrip1, and pCtIP proteins marked in green. No canonical Wwox binding motif is observed in the mouse or human Brca1 BRCT region.

The WW1 domain of human Wwox interacts with human Brca1 amino acid (aa) 305-1292 that exhibits the potential WW1 binding motif at Brca1-^981^PPLF^984^ [7] upstream of aa S988, which is phosphorylated by pChk2, and is critical for DSB repair pathway choice [16,38]. To determine if this exon 11 Wwox-binding motif is conserved in mouse Brca1, we aligned Brca1 aa sequences of different vertebrate species. The ^981^PPLF^984^ Brca1 sequence is conserved in primates but not in rodent species, though the mouse pChk2 phosphorylation site at aa S971 is conserved (Figure 1A) (canine and bovine species also do not show a known Wwox binding motif in this position, not shown). To test for Wwox-Brca1 interaction in mouse cells, WT MEFs were transiently transfected with Myc-Wwox, interacting proteins pulled down using Myc beads, and immunoblotted with Brca1 antiserum. Despite the lack of the PPLF motif, the Myc-Wwox protein co-immunoprecipitated Brca1, both unphospho- and hyperphosphorylated forms (Figure 1B). To determine where Wwox binds in mouse Brca1, we performed co-immunoprecipitation (co-IP) using MEF cells expressing mutant Brca1. Although murine Brca1 exon 11 does not exhibit a Wwox-binding motif, to rule out the possibility that Wwox binds through exon11, we tested Wwox-Brca1 interaction in mouse MEFs with exon 11 deleted (*Brca1^Δex11^*^/*Δex11*^) [39]. The mutant Brca1^Δex11^ binds to Myc-Wwox, confirming that the exon 11 region is dispensable for the mouse Brca1-Wwox interaction (Figure 1C). Next, to consider a contribution of the Brca1-BRCT repeats to Wwox binding, we performed co-IP experiments, using MEFs expressing a cancer-associated missense variant M1717R (*Brca1^M1717R^*^/*M1717R*^, equivalent to human Brca1 M1775R) located in the BRCT domain, that disrupts Brca1-BRCT complex formation with pAbraxas, pBrip1, and pCtIP [28,34]. WT Brca1 efficiently interacted with Myc-Wwox, whereas the Brca1-BRCT^M1717R^ mutant protein did not (Figure 1D). Furthermore, in situ association of Wwox-Brca1 is markedly decreased in another BRCT domain mutant cell line, *Brca1^S1655F^*^/*S1655F*^, as measured by PLA (Supplementary Figure S1), indicating that the WT mouse Brca1-BRCT tandem domain is required for Wwox-Brca1 interaction and this interaction is eliminated by these point mutations. 

### 2.2. Wwox Interaction with Brca1-BRCT Is Independent of Binding of Abraxas, Brip1, and CtIP

It is known that Brca1 is involved in HR and that its C-terminal tBRCT domain directly interacts with pAbraxas, pBrip1, and pCtIP. These three phospho-proteins participate in specific, timed steps in HR repair, while contributing to the stability of the genome [19,40]. Because the BRCT domain of Brca1 with the point mutations M1717R or S1655F is unable to bind the A, B, C complex proteins, it is likely that these mutations cause major disruption in the folded structure of the tBRCT domain [28,40]. Since our result suggests that Wwox binds to the tBRCT domain of Brca1 in mouse cells, we considered two possibilities, direct Brca1-BRCT-Wwox binding or indirect binding through Wwox-interaction with one of the Abraxas, Brip1, and/or CtIP proteins. Thus, we examined the aa sequence of mouse and human Brca1-BRCT domains for potential Wwox-binding motifs [41]. We did not find a known Wwox interaction motif in the murine or human Brca1-BRCT domain (Figure 1F). However, the evolutionarily highly conserved Wwox gene encodes a ubiquitous protein participating in a myriad of important cellular pathways to which literally hundreds of direct and potentially indirect binding partners have been described, with mechanisms and interaction motifs of the majority not defined [41]. To determine if Wwox directly binds to the tBRCT domain, not through interaction with Abraxas, Brip1, CtIP proteins, we performed Wwox-Brca1 co-IP in triple A, B, C mutant MEFs (*Abraxas^S404A^*^/*S404A*^; *Brip1^S994A^*^/*S994A*^; *CtIP^S326A^*^/*S326A*^), referred to as ABC mutant cells [34]. In ABC mutant cells, none of the Abraxas, Brip1, or CtIP proteins are able to interact with Brca1, due to abrogation of their phosphorylation sites [33,34,42]. Therefore, if one of the Abraxas, Brip1, or CtIP proteins function as a linker between Wwox and Brca1, Wwox-Brca1 interaction would be disrupted in ABC mutant cells. Instead, we observed that, in the ABC mutant cells, Wwox protein does bind to the Brca1-BRCT domain (Figure 1E), suggesting that the A, B, and C complex formation through Brca1-BRCT domain is unnecessary for Wwox-Brca1-BRCT interaction in mouse cells.

### 2.3. Brca1 Independent Interaction of Wwox with Abraxas, Brip1 and CtIP

**Figure 2:**
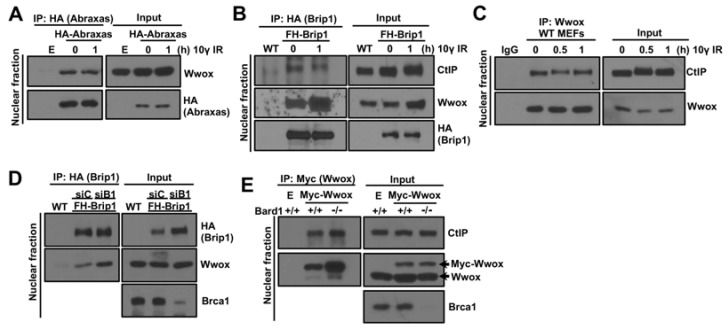
Wwox interaction with Abraxas, Brip1, and CtIP is independent of its binding to Brca1. (**A**) Murine Abraxas of the A complex binds to Wwox. HA-Abraxas plasmids were transfected into wild type (WT) MEFs and pulled down with HA-magnetic beads. Empty vector (**E**) transfected WT cells were used for negative control; (**B**) Murine Brip1 of the B complex binds to Wwox, as shown by its co-IP of Wwox and CtIP protein. IP was performed in MEFs expressing Flag-HA tagged wild type Brip1 protein (FH-Brip1). Brip1 protein was pulled down with HA beads and immunoblotted with CtIP and Wwox antisera. WT MEFs that do not express FH-Brip1 were used for negative control for HA pull-down; (**C**) Wwox interacts with CtIP in WT MEFs; (**D**) Wwox-Brip1 interaction is Brca1-independent. Wwox-Brip1 interaction occurred in both siControl (siC) and siBrca1 (siB1) transfected FH-Brip1 MEFs. Wild type MEFs (WT) that do not express FH-Brip1 served as negative control for HA pull-down; (**E**) Wwox-CtIP interaction is Brca1-independent. IP was performed in *Bard1^+/+^; 53BP1^−/−^* (labeled Bard1 +/+) and *Bard1^−/−^; 53BP1^−/−^* (labeled Bard1 −/−) MEFs. Wwox-CtIP binding occurred in the *Bard1^−/−^; 53BP1^−/−^* cells where Brca1 protein is unstable (**E**: empty vector transfection).

As we considered whether Wwox associated with a ligand that binds to the Brca1-BRCT domain, we performed co-IPs of Wwox with Abraxas, Brip1, and CtIP. To examine the Wwox-Abraxas interaction, we transiently transfected mouse HA-Abraxas into WT MEFs, pulled-down HA-Abraxas with HA magnetic beads, immunoblotted with HA and Wwox antisera, and observed that mouse Abraxas co-immunoprecipitated with Wwox in MEFs, and IR treatment did not affect the binding level (Figure 2A). We then tested for a Brip1-Wwox interaction in *Brip1^FHWT/FHWT^* MEFs [40] expressing FLAG-HA tagged Brip1 (FH-Brip1, given lack of a suitable Brip1 antiserum for MEFs). Interestingly, co-IP showed binding of Wwox to Brip1, with the interaction possibly enhanced by IR treatment (Figure 2B). Recently, a role for Brip1 in end-resection in cooperation with CtIP was reported in which Brip1-CtIP interaction was observed only after inducing DSBs [43]. In our mouse models, Brip1 interacts with CtIP with and without IR (Figure 2B). We also observed Wwox-CtIP interaction in WT MEFs (Figure 2C). To determine if Wwox-Brip1/CtIP binding is due to Brca1-BRCT interaction with Brip1/CtIP, we tested Wwox-Brip1 binding in Brca1-silenced FH-Brip1 MEFs (Figure 2D) and Wwox-CtIP binding in *Bard1^−^*^/*−*^; *53BP1^−/−^* MEFs (Figure 2E). Brca1 and Bard1 form a heterodimeric complex, and thus Brca1 stability in cells lacking Bard1 is dramatically reduced [44]. Our IP results showed that Wwox-Brip1/CtIP interactions are independent of Brca1, suggesting those three proteins are in a complex. In addition, Wwox binding to Abraxas, Brip1 and CtIP proteins, as well as to the Brca1-BRCT domain, may cooperatively regulate Brca1-A, B, C complex formation, possibly by blocking their phosphorylation. 

### 2.4. Effect of Wwox Loss on Timing of Appearance of Abraxas, Brca1 and CtIP Proteins at IR-Induced DSB Foci

The Brca1 tandem BRCT repeat domain forms a groove as a mutually exclusive binding site for pAbraxas, pBrip1, or pCtIP [27,45]. These Brca1 binding complexes are designated Brca1-BRCT A, B and C and are involved in the Brca1 repair pathway downstream functions [20,21,24,40]. We wondered if Wwox binding to the Brca1-BRCT domain or to A, B, C proteins themselves could influence recruitment of Abraxas, Brca1, or CtIP proteins to DSBs. To determine if the absence of Wwox protein expression in the Wwox KO MEF cells affects the timing of appearance of the Brca1-associated complex proteins that control execution of HR repair functions at IR-induced DSBs, we performed time-course studies to quantify Abraxas, Brca1, and CtIP foci at the DSBs in WT vs. KO MEFs after 10 γ IR. 

**Figure 3:**
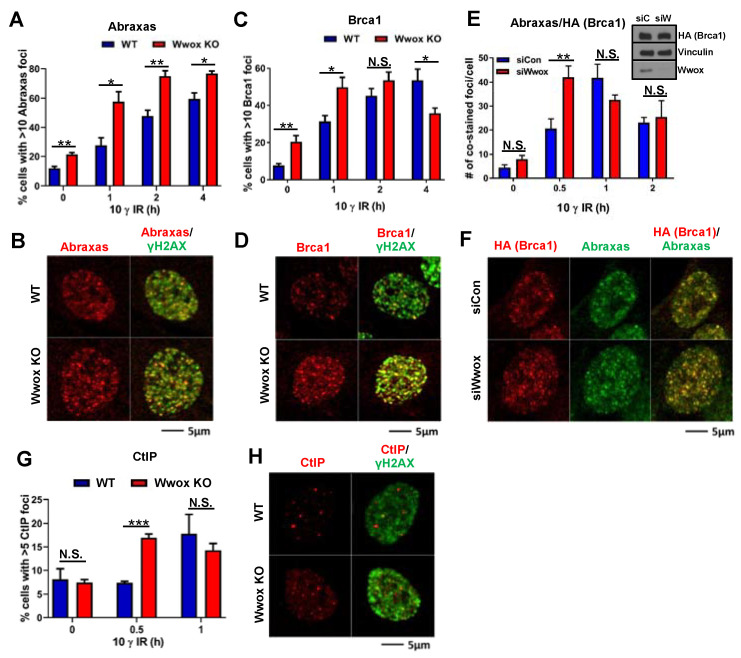
Timing of Abraxas, Brca1, CtIP of appearance in IR-induced foci in mouse MEFs (**A**–**D**) IR-induced focus formation of Abraxas (**A**,**B**) and Brca1 (**C**,**D**) was measured in WT and Wwox KO MEFs. Cells were irradiated (10 γ) and fixed at the indicated times. Wwox KO cells showed significantly more Abraxas foci positive cells at each time-point. An increase of Brca1 foci positive cells was observed in Wwox KO cells at 0 and 1 h post-IR, and no significant difference in WT vs. Wwox KO at 2 h post-IR. The number of Brca1 positive cells in Wwox KO cells was decreased at 4 h post-IR; representative images of Abraxas (red), Brca1 (red) and γH2AX (green) were from 1 h time-point (**B**,**D**). (**E**,**F**) Time-course study of colocalization of Abraxas with Brca1 foci in MEFs expressing Flag-HA tagged wild type Brca1 protein. Transient silencing of Wwox increased the co-stained number of foci at 0.5 h post-IR. Wwox silencing was confirmed by immunoblot (siC: siControl and siW: siWwox); representative images showing expression of Abraxas (green) and HA-Brca1 (red) were from 0.5 h post-IR (**F**). (**G**,**H**) CtIP foci formation is increased in Wwox KO cells at early time-point (0.5 h post-IR), but no significant difference was observed at 1 h post-IR. Error bars representing S.E.M and *p*-values were calculated by unpaired *t*-test (* *p* < 0.05, ** *p* < 0.01, *** *p* < 0.001); N.S., not significant. Experiments were carried out using two different wild-type (WT3, WT4) and KO (KO3, KO5) clones in duplicate.

As shown in Figure 3A, Abraxas foci in Wwox KO cells significantly exceeded the percentage of cells with >10 foci in WT cells at each time-point up to 4 h (Figure 3A, 1 h post-IR representative image shown in Figure 3B). Brca1 positive cells with >10 foci in KO cells exceeded those in WT cells at 0 and 1 h time-points, at 2 h the difference was not significant, at 4 h the WT cells show more Brca1 positive cells than Wwox KO cells (Figure 3C, 1 h post-IR representative image shown in Figure 3D). We also compared complexed Abraxas-Brca1 proteins at DSBs using *Brca1^FHWT^*^/*FHWT*^ cells [46], with or without Wwox silencing. In siControl transfected cells, the number of Abraxas-Brca1 co-localized foci was increased at 0.5 h, reached a peak at 1 h, and was decreased at 2 h after IR. In Wwox-silenced cells, the number of co-localized foci reached a peak at 0.5 h and decreased over time (Figure 3E, 0.5 h post-IR representative image shown in Figure 3F). Our results imply that Wwox binding to Brca1-BRCT domain proteins delays Abraxas-mediated Brca1 recruitment to foci. Therefore, loss of Wwox facilitates Abraxas-mediated Brca1 recruitment to DSBs. 

Brca1-mediated HR repair is initiated by DNA end-resection at DSBs, which generates 3′ ssDNA that can invade the homologous DNA strand. Thus, end-resection is a critical point for DSB repair pathway choice. It has been reported that CtIP is a key protein that induces a DNA nick to initiate end-resection by the MRN complex (Mre11-Rad50-NBS1) [23,30,31,32,47]. We compared CtIP focus formation at early time points after IR treatment in Wwox WT vs. KO MEFs. The percentage of CtIP positive cells with >5 foci in KO cells exceeded those of WT cells at 0.5 h post-IR (Figure 3G, 1 h post-IR representative image shown in Figure 3H). To examine CtIP protein at DSBs, we performed a PLA assay using CtIP and γH2AX antisera. Consistent with the IF results, we observed a dramatic increase of PLA foci at 0.5 h post-IR in Wwox KO cells (Supplementary Figure S2). In WT MEFs, the number of PLA foci gradually increased in a time-dependent manner (Supplementary Figure S2).

### 2.5. Loss of Wwox Expression in Mouse Cells Supports Initiation of HR through Rapid Engagement of End-Resection

**Figure 4:**
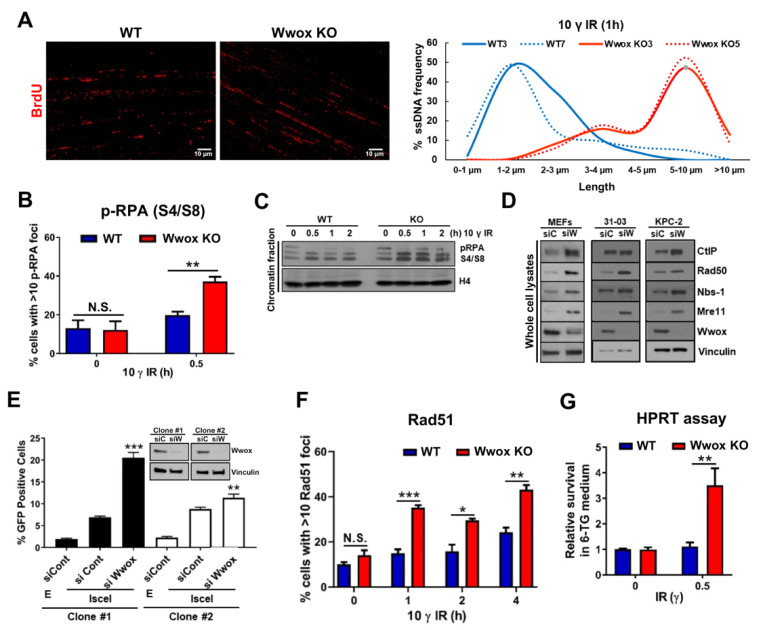
Wwox loss supports rapid initiation of HR through end-resection (**A**) Wwox expression delays the end-resection process. Plots of the ranges of ssDNA length vs. resected ssDNA frequency calculated by SMART assay. Long resected ssDNAs (5–10 μm) are detected in Wwox KO MEFs 1 h post-IR (10 γ) vs. the shorter ssDNA (1–2 μm) present in WT cells. The left panel shows representative images of ssDNA stained by BrdU 1 h post-IR (10 γ). (**B**) Immunofluorescence of pRPA in WT and Wwox KO MEFs show more pRPA recruitment in the KO cells at 0.5 h after 10 γ IR. Experiments were carried out using two different wild-type (WT3, WT4) and Wwox KO (KO3, KO5) clones in duplicate; (**C**) Immunoblot showing pRPA recruitment into chromatin fraction in Wwox KO vs. WT MEFs after IR. (**D**) Wwox silencing increases steady-state levels of Brca1-C complex proteins including CtIP, Rad50, Nbs1, and Mre11 in three different mouse cells (immortalized MEFs, mammary tumor (31-03) and pancreatic tumor cells (KPC-2)). (**E**) Wwox silencing enhances HR repair following ISce-1-induced DSBs in MEF/DR-GFP reporter cells. Bar graph shows % cells positive for GFP as an indication of relative HR, with corresponding immunoblots of cells carrying HR reporter, DR-GFP, in cells silenced for Wwox expression. (**F**) IR-induced Rad51 foci are increased in Wwox KO MEFs. Experiments were carried out using two different wild-type (WT3, WT4) and KO (KO3, KO5) clones in duplicate. (**G**) DSB repair in Wwox negative cells increases mutation acquisition. Frequency of mutations of the HPRT gene is increased in Wwox KO vs. wild-type WT MEFs following IR exposure. Survival data for the two WT and two KO clones are presented as averages. Error bars represent S.E.M and *p*-values were calculated by an unpaired t-test (* *p* < 0.05, ** *p* < 0.01, *** *p* < 0.001); N.S., not significant.

To determine if the absence of Wwox expression influences DNA end-resection, an essential early step for the HR pathway of DSB repair, we measured resection lengths at the level of individual DNA fibers by SMART assay in WT and KO MEFs (Figure 4A). ssDNA lengths at 1 h were significantly longer in IR-treated KO than WT MEFs, with long resected ssDNAs (5–10 μm) constituting ~50% of measured fibers, while ssDNAs are still short in WT cells (1–2 μm) (Figure 4A), indicating that Wwox absence accelerates the DNA end-resection process. Consistent with the SMART assay result, IR induced focus formation of pRPA32-S4/S8, a ssDNA marker, was elevated in Wwox KO vs. WT cells at 0.5 h post-IR (Figure 4B). In addition, pRPA recruitment to chromatin fraction was dramatically increased in Wwox KO cells after IR treatment (Figure 4C).

MRN complex (Mre11-Rad50-NBS1) and CtIP are crucial for promoting resection activity at the point of commitment to the HR repair pathway [23,30,31,32]. As Wwox expression has a role in attenuating the end-resection process, we asked if Wwox could modulate the level of MRN/CtIP complex proteins that interact with mouse Brca1. Thus, we silenced Wwox expression in three different mouse cell lines and measured the steady-state levels of CtIP and individual components of the MRN complex. As shown in Figure 4D, Wwox knockdown upregulated the basal levels of Brca1-C complex proteins vs. the siControl transfected cells, implying that Wwox regulates steady-state levels of complex C proteins (Figure 4D). Since these proteins are required for end-resection during HR repair, our data support the proposal that Wwox modulates the timing of recruitment of the C complex proteins to DSBs following IR exposure. 

Next, to determine if the elevated end-resection in Wwox deficient cells affects the efficiency of HR, we employed HR repair pathway reporter assays. In these assays, HR efficiency is quantified by expression of a GFP gene that has been correctly repaired via HR following induction of DSB at an ISce1 site. We transfected MEFs carrying this construct with siControl or siWwox oligonucleotides. As shown in Figure 4E, in Wwox-silenced cell clones, the absence of Wwox protein enhanced HR activity vs. siControl clones, which is consistent with our previous observation in human reporter cells [7]. We also assessed Rad51 focus formation by IF detection of foci following IR. Both WT and Wwox KO cells exhibit some RAD51 foci without IR treatment, with more Rad 51 foci in the KO vs. WT cells, suggesting that the Wwox absence affects basal activity of the HR repair pathway. Within 1 h of IR treatment, Rad51 foci were significantly increased in Wwox-deficient clones, with twice as many cells displaying >10 foci vs. WT clones. Unlike WT MEFs, the percentage of KO cells with >10 foci was slightly decreased by 2 h. Rad51 foci then gradually increased in both WT and KO cells and concordantly reached their peak levels by 4 h post-IR (Figure 4F). This elevated number of Rad51 positive cells upon IR treatment is also observed in Wwox silenced MEFs (Supplementary Figure S3).

Since our initial observations in MEFs established a genome caretaker function for Wwox [7], we next investigated whether Wwox could suppress mutation acquisition by assessment of colonies surviving in 6TG supplemented medium due to HPRT mutation, as a marker of mutant accumulation in the Wwox-deficient cells. The mutation frequency was increased in IR-treated KO MEFs, with WT cells acquiring 3.5-fold fewer colonies with HPRT mutations than KO cells (Figure 4G), likely due to inappropriate repair pathway choice. Results of these experiments collectively confirm that Wwox loss dysregulates DSB repair pathway choice after DSB induction by promoting activation of the HR pathway through immediate end-resection post-IR. 

### 2.6. Inhibition of End-Resection by Chk2 Silencing Sensitizes Wwox KO Cells to DNA Damaging Agents

Initiation of HR and SSA pathway choice is dependent on DNA end-resection. Our previous study found that Wwox deficiency in human cells treated with IR resulted in early DNA end-resection to tip the initial repair pathway choice toward HR and SSA, leading to resistance to DSB-inducing agents, IR, and cis- or carboplatin [7]. 

**Figure 5:**
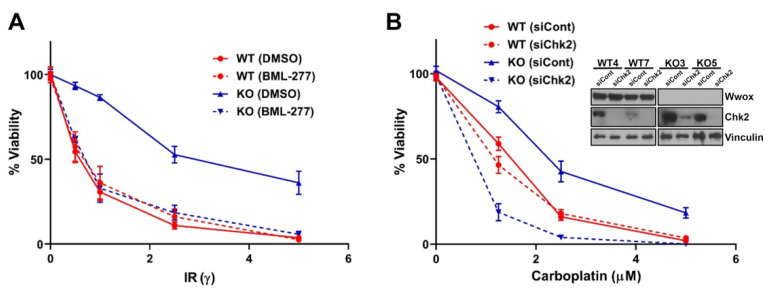
Inhibition of Brca1-associated HR sensitizes Wwox KO MEFs to DNA damaging agents. (**A**,**B**) Abrogation of Chk2 expression sensitizes Wwox KO MEFs to IR and carboplatin. Line graph depicting % survival of MEFs following exposure to IR or carboplatin with and without Chk2 inhibition by BML-277 treatment (**A**) or silencing (**B**). Wwox-deficient cells (blue solid line) exhibit increased survival vs. WT cells (red solid line). Chk2 inhibition or silencing confers IR and carboplatin sensitivity, respectively, in Wwox KO MEFs (blue dashed line) vs. WT (red dashed line).

It is well documented that Brca1 activation requires its phosphorylation by ATM/ATR and pChk2 [14,37]. Blocking pChk2 phosphorylation of Brca1 at S988 in human, S971 in mouse, and delayed end-resection at DSBs [38]. Thus, we hypothesized that delay of the early occurring end-resection in Wwox KO cells by inhibition of Brca1 phosphorylation by Chk2 could sensitize the Wwox KO cells to DNA damaging agents. To test this idea, we pretreated cells with Chk2 selective inhibitor, BML-277, or silenced Chk2 expression before IR or carboplatin treatment, and measured sensitivity by clonogenicity assays. Pretreatment with BML-277 dramatically reduced relative survival of Wwox KO cells after IR (Figure 5A). Similarly, Chk2 silenced Wwox KO MEFs were more sensitive to carboplatin treatment (Figure 5B). However, both Chk2 inhibitor treatment and Chk2 silencing did not impact relative survival in WT cells (Figure 5A,B). This selective killing effect on Wwox-deficient cells by combination treatment of IR/Carboplatin and Chk2 inhibitor was also observed in human cells [10], suggesting that inhibition or delay of Brca1 phosphorylation at S971 (S988 in human) by Chk2 inhibitor sensitize Wwox negative cells. Thus, concurrent treatment of IR/carboplatin with Chk2 inhibitor could be an alternative therapy to selectively target the high percentage of Wwox negative, treatment resistant tumors. 

## 3. Discussion

In our initial study of Wwox in human breast cancer cells [7], we reported Wwox interaction with Brca1 and upregulated HR/SSA pathways in Wwox-deficient cells. We also noted that established mouse KO MEFs and Wwox-deficient normal breast cells responded to IR or cisplatin exposure similarly to human cancer cells, and showed resistance to killing relative to WT MEFs and Wwox-proficient breast cells, respectively [6,7]. Our current study of the role of murine Wwox in response to repair of DSBs induced by IR or platinum drugs has found that: Wwox interacts with the Brca1-BRCT domain; Wwox also binds to Abraxas, Brip1, and CtIP, independently of Brca1, suggesting two levels of Wwox regulation of Brca1-BRCT complex formation; Wwox-deficient murine cells showed increased IR-induced Abraxas, Brca1, and CtIP foci formation and premature long resected single-strand DNA, early after IR; Wwox deletion increased the basal level of Brca1-CtIP interaction and expression level of MRN-CtIP protein complex; the delay of premature end-resection by combining Chk2 inhibition with IR or carboplatin treatment successfully restored IR and platinum sensitivity in Wwox deficient cells in vitro.

Since mouse models carrying knockout genes for numerous tumor suppressor genes (including Brca1 and associated pathway genes), or expressing oncogenic transgenes, are frequently used as models to determine functions of novel gene products, their mechanisms of action, and for preclinical trials of new drug responses, it seemed important to continue our examination of mouse cell responses to DNA damage and DSB repair pathways. Thus, we further investigated the parallels observed in functions of Wwox and its absence in mouse DNA damage repair pathways. The implications of the new findings and the resulting new questions are discussed here.

### 3.1. The Murine Brca1-BRCT Tandem Domain Is Required for Binding of Wwox to Brca1

Wwox binds to the mouse Brca1-BRCT domain, independently of the Brca1 phospholigands, pAbraxas, pBrip1, and pCtIP. Murine Brca1-BRCT domain mutations (analogous to M1775R, S1655F in human, identified in familial breast cancers) abrogate not only binding of the A, B, C complexes to the BRCT domain, but also the BRCT-Wwox interaction, i.e., MEFs expressing the Brca1 S1598F mutation, are defective in HR repair and the homozygous mutant mice are highly susceptible to tumor development [40]. These findings indicate that Brca1-BRCT domain functions are severely negatively affected by single amino acid changes and that the effects include abrogation of interactions of the domain with Wwox and the protein complexes essential for DSB repair by the HR pathway. Thus, it does not seem surprising that binding of a protein such as Wwox (46 kD) to a region within this tandem repeat domain might also interfere with the BRCT domain folded structure and essential downstream functions. Therefore, we hypothesize that Wwox binding to the mouse BRCT domain delays recruitment of Brca1-BRCT associated repair proteins to DSBs by controlling the timing of A, B, C complex formation. 

### 3.2. Wwox Loss Accelerates Brca1-CtIP and MRN Complex Formation 

**Figure 6:**
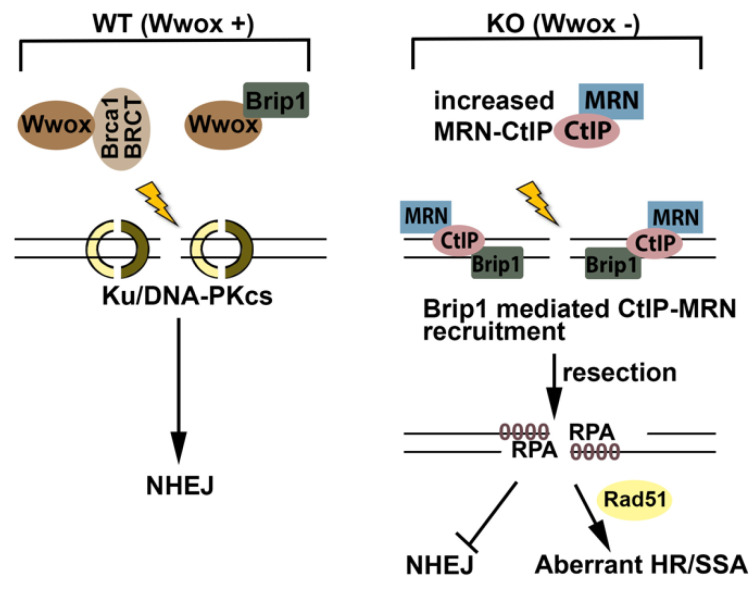
Proposed model illustrating Wwox role in repair of DSBs immediately following IR or platin exposure. In cells expressing Wwox, Wwox binding to BRCT blocks Brca1-BRCT complex formation and BRCA1 activation; Wwox-Brip1 interaction inhibits Brip1 mediated CtIP-MRN recruitment to DSBs. In the absence of Wwox, both Brca1-BRCT and Brip1 proteins are more accessible for interaction with CtIP and MRN complex even without IR. The pre-formed MRN-CtIP complex is rapidly recruited to DSBs by Brip1 and initiates resection immediately, which inhibits NHEJ and promotes HR/SSA repair. These repairs are mutagenic causing IR/Platinum resistance.

The Brca1-C complex has a role in end-resection. Reczek et al. have shown that ablation of Brca1-CtIP interaction did not affect pRPA foci formation after IR in murine cells [33], likely because, without binding to Brca1, the CtIP-MRN complex can localize to DSBs and promote resection through Brca1 independent Brip1-CtIP interaction [43]. Nevertheless, Brca1-CtIP interaction speeds up resection, and resection speed can be involved in repair pathway choices [35]. Wwox co-IPs with Brip1 and CtIP, and the bindings are independent of Brca1. We believe that Wwox binding to Brip1 and CtIP acts as a brake to inappropriate HR initiation by inhibiting binding of these complexes to the Brca1-BRCT domain and preventing immediate end-resection after IR. Thus, the Brip1 and CtIP binding and associated DNA resection at DSBs in Wwox-deficient cells are key events that determine the choice of HR repair immediately post-IR (Figure 6).

Unexpectedly, Wwox deficiency dramatically increases basal levels of Brca1-C complex components that play a key role in end-resection. In Wwox KO cells, the level of MRN complex and Brca1-CtIP binding is substantially increased, even without DNA damage induction, likely due to higher protein levels of MRN/CtIP complex components. It is worth noting that, in IR exposed WT cells, the percentage of cells containing CtIP positive foci gradually increased in a time dependent manner, but, in Wwox KO cells, we see that CtIP, essential for the MRN-induced nicking step that initiates DNA resection, is already at DSB foci at 0.5 h post IR. The increased MRN complex level may preset the cells to promote end-resection immediately after DNA damage and increase the resection speed in Wwox KO cells. Thus, the cells would initially repair DSBs only through end-resection mediated pathways, HR and SSA, which are upregulated in Wwox-deficient cells [7]. Expression levels of MRN complex components are correlated with worse prognosis and chemo/radiotherapy resistance [48,49,50], as observed in Wwox negative tumors [6,7]. Our results showed elevated protein levels of CtIP and MRN complex in both Wwox-silenced and KO MEFs, suggesting that Wwox expression level is useful for predicting therapeutic efficacy.

### 3.3. Wwox Absence in Mouse Cells, as in Human, Favors the Choice of HR Repair Early after DSB Induction

Wwox absence leads to very early DSB-induced DNA end-resection and enhances pRPA, Rad51, Abraxas, Brca1, and CtIP foci formation beginning at 0.5 h post IR in mouse cells and even earlier end-resection in human cancer cells [10]. The consequent early HR repair of DSBs associated with Wwox absence is significant since it enhances the subsequent resistance to commonly used DNA damaging drugs, such as IR and carboplatin, and confers a survival advantage to Wwox-deficient cells, as we have previously shown for human ovarian and lung cancer patients [6]. 

We have observed that Brca1 IRIF in Wwox KO cells reached a peak at an earlier time point (2 h, post-IR) than those in WT cells, and then were reduced at 4 h after IR. We propose that, when Brca1 is released from the DSBs by degradation, and new Brca1 is synthesized, NHEJ repair may begin, and normal regulation of repair could take over. Without synchronization of cells, which itself can cause DNA damage, study of this transition is difficult in cells where different repair pathways are underway in different subpopulations of cells. In accordance with this proposal, we expect that the DNA repair pathway choices are thus normalized, such that Wwox-deficient cells begin repairing remaining DSBs by all appropriate pathways, meaning that the early HR repair is aberrant but not all DSB repair is aberrant. Thus, the Wwox deficient cells, which occur in large fractions of many breast and ovarian cancers, are resistant to killing by these agents and these resistant cells survive with accumulation of some aberrantly repaired DSBs. These surviving mutant cells in a cancer patient lead to growth of cancers that will be progressive following carboplatin treatment. 

How and why does absence of Wwox expression, which occurs so frequently in many types of cancers, dysregulate the HR repair pathway so dangerously, or put another way, why do cells need a Wwox ‘brake’ for end-resection? Without Wwox, end-resection, a critical process to determine DSB repair pathways, occurs immediately, which inhibits NHEJ repair, the dominant DSB repair pathway. We are proposing that the normal function of Wwox, in the context of DSB repair in mouse, binds to the Brca1-BRCT tandem domain, as well as to A, B, and C proteins and inhibits formation of the Brca1-BRCT complexes, delaying initiation of end-resection at DSBs, as in Figure 6, illustrating our proposed model for Wwox function in DSB repair. Wwox binding to murine Brca1-BRCT may alter the tandem BRCT domain structure such that the phospho Abraxas, Brip1, and CtIP protein complexes do not immediately bind to Brca1-BRCT protein. Wwox-Brip1 binding would thus synergistically block the Brip1 and CtIP proteins from interaction with Brca1.

We have shown that inhibiting early occurring end-resection by Chk2 inhibition successfully killed Wwox-deficient MEFs by causing synthetic lethality, but did not affect viability of WT MEFs, suggesting that IR or platinum-based therapy, combined with inhibition of Chk2 activation could be a useful strategy to target Wwox-negative, treatment resistant cancers. Although we have not yet confirmed all aspects of the mechanisms by which Wwox executes this modulatory influence to prevent premature activation of HR, we hypothesize that generation of murine cells expressing mutations or deletions in the potential Wwox-binding motifs would lead to the phenotypes observed in this study in Wwox knockout or silenced cells.

## 4. Materials and Methods

### 4.1. Cell Lines and Transfection

All cells were cultured in high glucose DMEM supplemented with 10% heat-inactivated fetal bovine serum (FBS), 100 units penicillin/100 μg/mL streptomycin at 37 °C in 5% CO_2_. Designated wild type (WT) and KO MEFs (WT3, WT4, WT7, KO3, and KO5) were isolated from individual mouse embryos as described before [7]. Mutant MEFs immortalized by SV40 large T antigen transfection were: *Brca1^+^*^/*+*^; *Pim^DRGFP^*^/*+*^ [51], *Brca1^Δex11^*^/*Δex11*^ [39], *Brca1^S1598F^*^/*S1598F*^ [40], *Brca^M1717R^*^/*M1717R*^ [34], *Brca1^FHWT^*^/*FHWT*^ [46], *Brip1^FHWT^*^/*FHWT*^ (referred to as FH-Brip1 in figure) [40], *Abx^S404A^*^/*S404A*^; *Brip1^S994A^*^/*S994A*^; *Ctip^S326A^*^/*S326A*^ (carrying mutations of the serine residues in the pSPXF motifs of each of these A, B, C proteins, referred to as ABC mutant cell line) [34], *Bard1^+/+^; 53BP1^−/−^*, *Bard1^−/−^*; *53BP1^−/−^*, mouse mammary tumor cells (31-03, *p53^LSL-R270H/+^*; *Wap Cre*) [40], and mouse pancreatic tumor cells (KPC-2, *Kras^G12D/+^*; *p53^R270H/+^*; *Pdx1-Cre*) [52] were obtained from the laboratory of Dr. Thomas Ludwig (Department of Cancer Biology and Genetics; Ohio State University; Columbus, OH, USA). The plasmids Myc-DDK-tagged mouse Wwox (Cat# MR206526, Origene; Rockville, MD, USA), HA-mouse CtIP (Cat# EX-Mm23321-M06, GeneCopoeia^TM^; Rockville, MD, USA), and siRNA for silencing (siControl-Cat# D-001810-01, siWwox-Cat# L-063111-01, siBrca1-Cat# L040545-00, and siChk2-Cat# L040604, Dharmacon; Lafayette, CO, USA) were transfected with the JetPEI transfection reagent (Cat# 101-10N, Polyplus; Illkirch, France) following manufacturer protocols. Cell lysates were collected 48 h post-transfection for western blot and IP analyses.

### 4.2. Immunofluorescence Staining and Duolink In Situ Proximity Ligation Assay (PLA)

MEF cells were seeded on poly-L-lysine coated glass coverslips. After IR (10 γ) treatment, cells were washed with PBS, and fixed in 4% paraformaldehyde for 15 min. For CtIP staining, cells were incubated in cold extraction buffer (10 mM Pipes pH 6.8, 100 mM NaCl, 300 mM sucrose, 3 mM MgCl_2_, 1 mM EGTA, 0.5% Triton X-100) for 5 min before fixation in 4% paraformaldehyde. Fixed cells were then permeabilized in 0.2% Triton X-100 (10 min), blocked by 5% bovine serum albumin in PBS for 1 h, and incubated with specific primary antisera in a humidified chamber. After washing in PBS-T (0.1% Tween in PBS), cells were incubated with secondary antibodies conjugated to Alexa Fluor for 1 h in a humidified light-resistant chamber. After washing in PBS-T, coverslips were incubated in Hoechst 33342 for nuclei visualization, and mounted with Aqua-Poly/Mount medium and dried overnight. PLA staining was performed according to manufacturer’s protocol (Sigma; St. Louis, MO, USA). For focus counting, images were acquired by Zeiss Axioskop 40 microscope with 40× objective lens and foci manually quantified. Representative images were acquired by confocal microscope (Olympus Spectral FV1000, Olympus; Tokyo, Japan) with 100× objective lens. Bar graphs of the data represent quantification of averages of foci numbers of three independent experiments, generated using Graph-Pad Prism 7 software. 

### 4.3. Western Blot and Immunoprecipitation Assays 

Cells were lysed in either cold 1× RIPA buffer or low salt NP40 lysis buffer (10 mM Hepes, pH 7.6, 0.25 M NaCl, 0.1% NP40, 5 mM EDTA, 10% Glycerol) supplemented with Protease Cocktail Inhibitor tablet (Roche; Basel, Switzerland). Proteins were separated on 6–12% SDS-PAGE gels and transferred onto nitrocellulose membranes (Amersham; Amersham, UK). Membranes were blocked with 5% skim milk in TBS-T (150 mM NaCl, 250 mM Tris-HCl pH 7.4, 0.01% Tween 20) followed by overnight incubation with specific primary antisera at 4 °C. Upon washing with TBS-T, the HRP-conjugated secondary antisera were added for an hour at room temperature. After washing with TBS-T, immunoreactive proteins were detected using SuperSignal^TM^, West Pico PLUS Chemiluminescent Substrate (Cat# 34579, Thermo Fisher Scientific; Waltham, MA, USA). For IP, cells were harvested at 48 h after transfection, and 500 µg whole cell lysates or soluble nuclear extract were incubated with 20 μL Myc or HA-magnetic beads, overnight at 4 °C. After multiple washes, protein complexes were eluted by boiling the beads for 5 min in 15 μL of 2X reducing sample buffer, and precipitates separated on SDS-PAGE as described above.

### 4.4. Isolation of Soluble Nuclear Fraction 

Cells were harvested in nonnuclear extraction buffer (0.5% NP-40, 10 mM HEPES pH 7.9, 10 mM KCl, 0.1 mM EGTA, 0.1 mM EDTA and protease inhibitors), incubated on ice for 15 min, centrifuged at 5000× *g* for 5 min, and supernatants collected for the nonnuclear fraction. Pellets were washed in nonnuclear extraction buffer and incubated in low salt NP40 lysis buffer for 30 min on ice, centrifuged at 16,000× *g* for 10 min, and the supernatants were collected as the soluble nuclear fraction. 

### 4.5. DR-GFP Assay 

Green fluorescence protein (GFP) reporter assays were performed using two independent MEF clones carrying the DR-GFP reporter construct in the Pim1 locus as described previously [51]. Briefly, I-SceI or empty vector were transfected by electroporation using Mirus Ingenio Solution (Mirus Bio; Madison, WI, USA), and cells were harvested 48 h after transfection. Cell pellets were resuspended in 1% FBS in PBS and transferred to 10 cm plates; after 48 h, the percentage of GFP positive cells was quantified by flow cytometry (BD^TM^ LSR II).

### 4.6. Chemicals and Antisera

Carboplatin (AdipoGen, San Diego, CA, USA, AG-CR1-3591) and BML-277 (SelleckChem, Houston, TX, USA, Cat#S8632) were dissolved in water (10 mg/mL) and in dimethyl sulfoxide (10 mM), respectively, and stored at 20 °C. Further dilutions of each drug for the respective experiments were freshly prepared in the growth medium. Table 1 lists antisera and dilutions used for various experiments.

### 4.7. Single Molecule Analysis of Resection Tracks (SMART) Assay

To measure the length of resected single-strand DNA after IR, we used the SMART assay [35]. Wwox WT and KO MEFs were grown to ~70% confluency and labeled with BrdU (25 μM) overnight. BrdU incubated cells were treated by IR (10 γ), trypsinized, centrifuged, and resuspended in 200 μL PBS (10^6^ cells/mL) at 1 h; 4 μL of cell suspension was spotted onto the edge of a pre-marked glass slide, and allowed to air dry 5 min. The air-dried cells were mixed with 5 μL lysis buffer (0.5% SDS, 200 mM Tris-HCl, pH 7.4, 50 mM EDTA) on glass slides. After 5 min, an additional 10 μL of lysis buffer was added and slides were immediately tilted (~15°) to spread DNA fibers by gravitational flow. Slides were then fixed using 3:1 methanol/acetic acid for 10 min. Excess solution was drained off and fixed slides were air-dried. Slides were washed in PBS, blocked in 1% BSA in PBS and incubated with BrdU antiserum (1:100, ab6326, Abcam; Cambridge, England) for 1 h. After washing in PBS, slides were incubated in anti-Rat Alexa fluor 594 (1:500, ab150160, Abcam) and unbound antiserum removed by PBS washes. DNA fibers were stained using Hoechst 33342 and mounted with Aqua-Poly/Mount medium. Images were acquired using Zeiss Axioskop 40 microscope with 40X objective lens, and non-tangled fibers were measured using ZEN2 software. Experiments were carried out using two WT (WT3, WT7) and two KO (KO3, KO5) clones.

### 4.8. Colony Formation and Mutation Assays

Colony assays were performed to compare sensitivity to IR and carboplatin in Wwox KO vs. WT MEF cells with and without Chk2 silencing or inhibition. Briefly, cells were treated with different doses of carboplatin (0, 1.25, 2.5, and 5 µM) and IR (0, 0.5, 1, 2.5, 5 γ) 24 h and 2 h before seeding, respectively. Cells were washed in PBS, trypsinized, counted, and 5000 cells plated in 60 mm culture dishes in triplicate, were grown for 10–14 days and fixed, stained with crystal violet, and colony numbers counted under microscope. Percent survival is expressed as the number of colonies in the IR-treated or carboplatin-treated condition at the indicated dose of drug vs. the untreated condition. The frequency of 6-thioguanine (6-TG) resistant colonies among WT or KO MEF cells assessed the frequency of hypoxanthine-guanine phosphoribosyl transferase (HPRT) mutant colonies. Cells were treated with low-dose IR (0, 0.5, 1 γ) and equally seeded (5000 cells) into 60 mm culture dishes in triplicate. Plated cells were cultured in medium containing 6-TG (Sigma, 5 μM) with medium replaced every 2–3 days. Cells were maintained at 37 °C and 5% CO_2_ for 2 weeks, stained by crystal violet, and colony numbers counted by microscopic observation. Survival (%) bar graph was plotted relative to colony numbers of non-treated control cells.

### 4.9. Statistical Analysis

Statistical analyses were performed using an unpaired two-tailed Student’s *t*-test to compare sets of results from independent groups, and values of *p* < 0.05 were considered statistically significant. Each bar graph included at least three replicates with error bars representing S.E.M and *p*-values calculated using Graph-Pad Prism 7 software.

## Figures and Tables

**Table 1:** Antisera Information.

Antisera	Source	WB	IP	IF/PLA
Brca1	57X from Ludwig Lab	1:2000		1:500
Abraxas	Ludwig lab	1:5000		1:3000
CtIP (mab)	11-1 from R. Baer Lab	1:50		1:25
CtIP	Santa Cruz, SC-271339	1:300		1:100
Rad51	Abcam, ab133534			1:500
Phospho RPA32 (S4/S8)	Bethyl, A300-245A			1:1000
Mre11	Cell signaling, #4895S	1:1000		
Rad50	Cell signaling, #3427S	1:1000		
Nbs1	Novus, NB100-143	1:2000		
Wwox	Huebner Lab	1:5000	1:125	
Vinculin	Invitrogen, 700062	1:5000		
HA	Roche, 11867423001	1:500		
BrdU	Abcam, ab6326	1:100		
Chk2	Cell signaling, #3440S	1:1000		
Gamma H2AX	Cell signaling, #9718S			1:500
Gamma H2AX	Sigma, #05-636			1:5000
HA magnetic beads	Pierce, 88837		20 μL	
Myc magnetic beads	Pierce, 88842		20 μL	

## Data Availability

All inhouse produced materials are available upon request. The flow cytometry data for the GFP reporter assay will be publicly available at Flow Repository upon publication of the manuscript.

## Supplementary Materials

The following supporting information can be downloaded at: https://www.mdpi.com/article/10.3390/ijms23073729/s1.
