# Supplementary material for: Wwox Binding to the Murine Brca1-BRCT Domain Regulates Timing of Brip1 and CtIP Phospho-Protein Interactions with This Domain at DNA Double-Strand Breaks, and Repair Pathway Choice
Source: Int J Mol Sci. 2022 Mar 28;23(7):3729. doi: 10.3390/ijms23073729 (PMC8999063; doi:10.3390/ijms23073729)
Supplement: Supplementary file 1 [file ijms-23-03729-s001.zip › ijms-1628093-supplementary.pdf]

SUPPLEMENTARY FIGURES

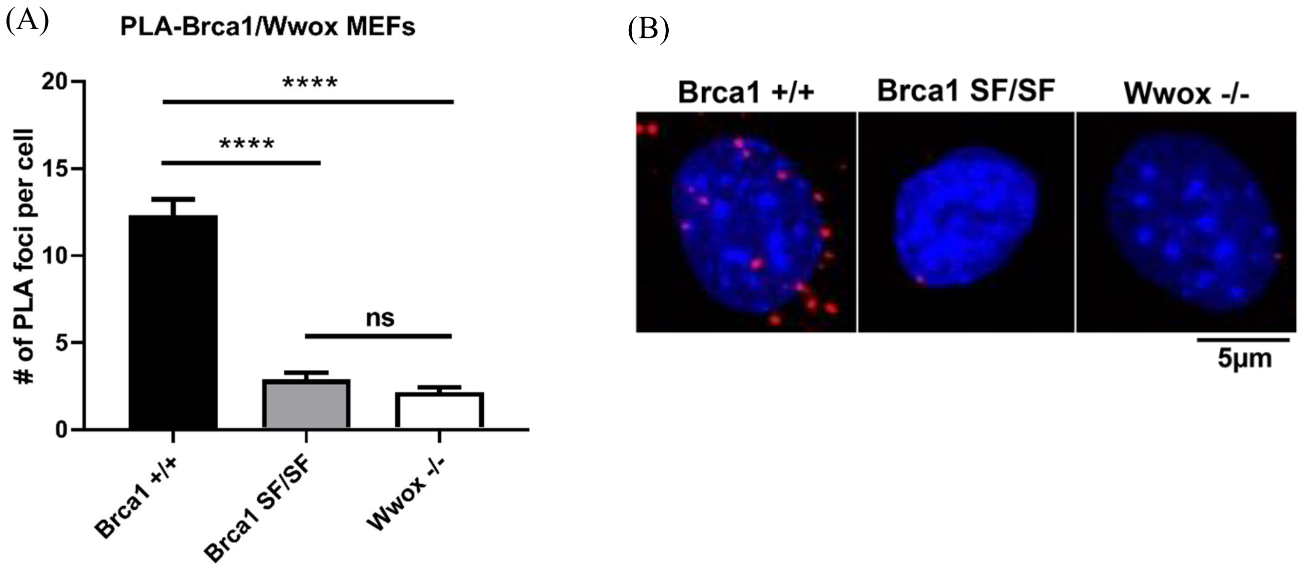

Figure S1. BRCT domain of murine Brca1 is crucial for Wwox-Brca1 interaction. Proximity Ligation Assay (PLA) (**A**) showed that Brca1-Wwox interaction does not occur in MEFs expressing BRCT domain mutant Brca1$^{S1655F/S1655F}$ (SF/SF). Error bars represent S.E.M (**B**) and p-values were calculated by one-way ANOVA test (**** p<0.0001).

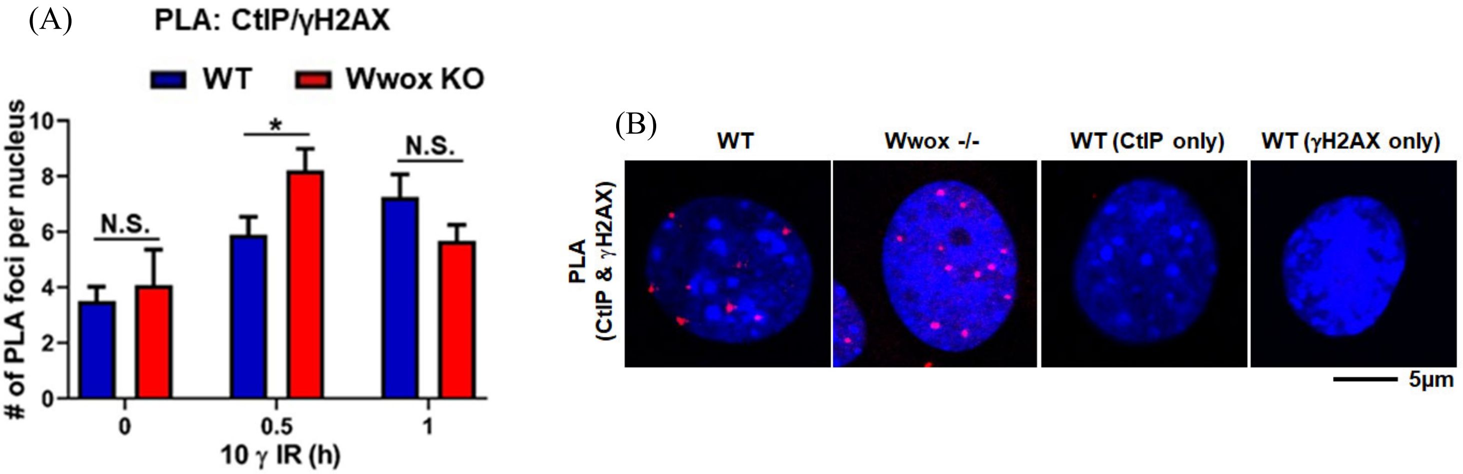

Figure S2. CtIP recruitment to DSBs occurs more dramatically in Wwox KO MEFs. In WT cells, the number of CtIP-γH2AX PLA foci gradually increased in a time dependent manner, while in KO, the number of PLA foci was more dramatically elevated at 0.5h after IR and decreased at 1h (**A**). Error bars represent S.E.M (**B**) and p-values were calculated by unpaired t-test (*p<0.05); N.S., not significant. The representative images shown are 0.5 h after IR.

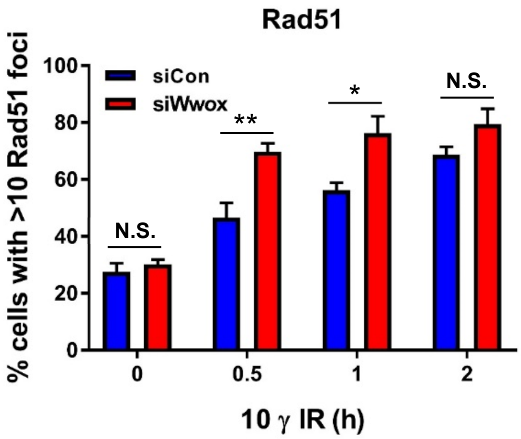

Figure S3. Wwox silenced WT MEFs have significantly more Rad51 positive foci than siControl (siCon) transfected WT MEF cells. Error bars represent S.E.M and p-values were calculated by unpaired t-test (*p<0.05, **p<0.01, N.S., not significant).
